# GABA tonic currents and glial cells are altered during epileptogenesis in a mouse model of Dravet syndrome

**DOI:** 10.3389/fncel.2022.919493

**Published:** 2022-07-21

**Authors:** Rosa Chiara Goisis, Angela Chiavegato, Marta Gomez-Gonzalo, Iacopo Marcon, Linda Maria Requie, Petra Scholze, Giorgio Carmignoto, Gabriele Losi

**Affiliations:** ^1^Department of Biomedical Science, University of Padua, Padua, Italy; ^2^Neuroscience Institute, National Research Council (IN-CNR), Padua, Italy; ^3^Department of Pathobiology of the Nervous System, Center for Brain Research, Medical University of Vienna, Vienna, Austria

**Keywords:** extrasynaptic GABA receptor A, delta subunits, GFAP - glial fibrillary acidic protein, Iba-1, SCN1A encephalopathy, Nav1.1 channel

## Abstract

Dravet Syndrome (DS) is a rare autosomic encephalopathy with epilepsy linked to Na_v_1.1 channel mutations and defective GABAergic signaling. Effective therapies for this syndrome are lacking, urging a better comprehension of the mechanisms involved. In a recognized mouse model of DS, we studied GABA tonic current, a form of inhibition largely neglected in DS, in brain slices from developing mice before spontaneous seizures are reported. In neurons from the temporal cortex (TeCx) and CA1 region, GABA tonic current was reduced in DS mice compared to controls, while in the entorhinal cortex (ECx) it was not affected. In this region however allopregnanonole potentiation of GABA tonic current was reduced in DS mice, suggesting altered extrasynaptic GABA_A_ subunits. Using THIP as a selective agonist, we found reduced δ subunit mediated tonic currents in ECx of DS mice. Unexpectedly in the dentate gyrus (DG), a region with high δ subunit expression, THIP-evoked currents in DS mice were larger than in controls. An immunofluorescence study confirmed that δ subunit expression was reduced in ECx and increased in DG of DS mice. Finally, considering the importance of neuroinflammation in epilepsy and neurodevelopmental disorders, we evaluated classical markers of glia activation. Our results show that DS mice have increased Iba1 reactivity and GFAP expression in both ECx and DG, compared to controls. Altogether we report that before spontaneous seizures, DS mice develop significant alterations of GABA tonic currents and glial cell activation. Understanding all the mechanisms involved in these alterations during disease maturation and progression may unveil new therapeutic targets.

## Introduction

Dravet Syndrome (DS) is a severe encephalopathy with epilepsy linked in most cases to *de novo* loss of function mutations of the SCNA1 gene encoding for the α subunit of Na_v_1.1 voltage-dependent sodium channels (Claes et al., 2001; Catterall et al., 2010; Dravet, 2011). These channels are mainly expressed on GABAergic interneurons, which leads to the hypothesis that impaired GABAergic signaling is the main cause of hyperexcitability in DS (Yu et al., 2006; Ogiwara et al., 2007). Na_v_1.1 heterozygous mice (DS for brevity) show a similar phenotype to DS patients including hyperthermia-induced seizures, ataxia, cognitive disorders, autistic-like behaviors, and spontaneous drug-resistant seizures (Yu et al., 2006; Oakley et al., 2009; Tai et al., 2014). Although interneuron excitability defect in DS is the most direct and well documented consequence of Na_v_1.1 haploinsufficiency, recent studies suggest that other mechanisms must be also involved. For instance in Na_v_1.1^+^^/-^mice significant changes of both inhibitory and excitatory synaptic currents (De Stasi et al., 2016) or both cellular excitability (Tran et al., 2020) are reported. Another study on brain extracts from adult DS patient autopsies reports altered expression of GABA_A_ receptors and intracellular chloride homeostasis (Ruffolo et al., 2018). Altogether these findings prompt for deeper studies focusing on the molecular changes in the overall GABAergic system and its interplaying partners in DS.

The complexity of GABA signaling derives from the high heterogeneity of interneuron cellular types (Rudy et al., 2011) and from the different GABA_A_ receptor subunit composition that confers specific functional and pharmacological properties to the GABA_A_ receptor channel complex (Mody and Pearce, 2004). Synaptic GABA_A_ receptors composed of α_1–3_, β_n_, and γ_2_ subunits mediate fast phasic currents, while extrasynaptic receptors composed of α_4–6_, β_n_, and δ subunits mediate slow tonic currents (Farrant and Nusser, 2005). Specifically, GABA tonic conductance is very important in regulating cellular and network excitability, in both physiological and pathological conditions, including epilepsy (Brickley and Mody, 2012). Altered tonic conductance has been reported in some forms of epilepsy, such as absence epilepsy (Cope et al., 2009) and temporal lobe epilepsy (Houser et al., 2013; Pavlov and Walker, 2013). Despite the importance of extrasynaptic inhibitory signals in setting brain circuit excitability, GABA tonic currents in DS remain largely unexplored. Using patch-clamp recordings from neurons in brain slice preparations and immunofluorescence techniques, we here investigated whether and how GABA tonic currents/extrasynaptic GABA_A_ receptors in mice are altered during DS epileptogenesis in areas that are important for seizure generation. Given the strong involvement of neuroinflammation in epilepsy (van Vliet et al., 2018; Vezzani et al., 2019; Pracucci et al., 2021) and its emerging role in genetic disorders characterized by seizures (Higurashi et al., 2015), we also evaluated whether reactive glial cells are present in the early phase of the disease maturation before overt seizures are reported.

## Materials and Methods

### Animals

All experiments involving animals were performed in accordance with the guidelines established by the European Communities Council Directive and approved by the National Council on Animal Care of the Italian Ministry of Health. All efforts were made to minimize the number of animals used and their suffering, following the “3Rs” principle (reduction, replacement, and refinement). The colony of Scn1a heterozygous mice carrying a Scn1a gene ablation with a replacement-type construct in the last coding exon (Yu et al., 2006) was maintained on CD1 background to reduce epileptic phenotype as in Liautard et al. (2013) and De Stasi et al. (2016). Experiments were performed on mice with mixed backgrounds (C57BL6:CD1) and genotyped as previously described (Liautard et al., 2013). We used heterozygous (Na_v_1.1^+^^/-^; DS mice) and wild-type (wt) mice same age littermates of either sex between postnatal days 15 and 19 (P15–P19), i.e., before spontaneous seizures are reported, i.e after P21 (Yu et al., 2006) or later than P22 (Oakley et al., 2009).

### Brain slice preparation and patch-clamp recordings

Coronal slices of 350 μm were obtained as in Sessolo et al. (2015). Animals were anesthetized as reported above, the brain removed and transferred into an ice-cold solution (ACSF, in mM: 125 NaCl, 26 NaHCO_3_, 2.5 KCl, 2 CaCl_2_, 1 MgCl_2_, 25 glucose, pH 7.4 with 95% O_2_ and 5% CO_2_). Slices were cut in the solution reported in Sessolo et al. (2015) and then kept for 1 min in the solution (in mM): 225 D-mannitol, 2.5 KCl, 1.25 NaH_2_PO_4_, 26 NaHCO_3_, 25 glucose, 0.8 CaCl_2_, 8 MgCl_2_ with 95% O_2_ and 5% CO_2_. Finally, slices were kept in ACSF at 30°C for 20 min and then maintained between 19 and 22°C for the entire experiment. For whole-cell patch-clamp recordings, slices were perfused in a submerged chamber at a rate of 3 ml min^−1^ with (in mM): 120 NaCl, 2.5 KCl, 1 NaH_2_PO_4_, 26 NaHCO_3_, 1 MgCl_2_, 2 CaCl_2_, 10 glucose, pH 7.4 (with 95% O_2_ and 5% CO_2_), and 2,3-dioxo-6-nitro-7-sulfamoyl-benzo[f]quinoxaline (NBQX, 10 μM), (2*R*)-amino-5-phosphonovaleric acid (APV, 50 μM) to block glutamate ionotropic receptors and Tetrodotoxin (TTX, 1 μM) to block action potential firing. Neurons or astrocytes were visualized under a confocal microscope (TCS-SP5-RS, Leica Microsystems, Germany) equipped with a CCD camera for differential interference contrast (DIC) image acquisition. Single cell recordings were performed in voltage- or current-clamp configuration using a multiclamp-700 B amplifier (Molecular Devices, USA). Signals were filtered at 1 kHz and sampled at 10 kHz with a Digidata 1440s interface and pClamp10 software (Molecular Devices, USA). The pipette resistance was 3–4 MΩ. Access resistance was monitored throughout the recordings and was <25 MΩ. Neurons that had *a* > 15% change in access resistance during the recording were discarded. Whole-cell intracellular pipette solution was (in mM): 70 K-gluconate, 70 KCl, 10 HEPES, 1 EGTA, 2 MgCl_2_, 4 MgATP, pH adjusted to 7.2 with KOH, 270–290 mOsm. With these extracellular and intracellular solutions, the GABAergic current is inward with a theoretical ECl of −16 mV. Recordings were performed at the holding potential of −70 mV. Data were not corrected for the liquid junction potential. Recordings were analyzed with Clampfit 10.6 (Molecular Devices). GABA tonic currents were evaluated by subtracting the mean current (from 30 s epochs) before and after GABA_A_ antagonist perfusion (Bicuculline 25 μM for all experiments except for older experiments in TeCx in which picrotoxin 100 μM was used). In a subset of experiments we alternatively measured the tonic current by fitting a Gaussian curve to the positive side of all-points histograms of the same epochs in control and after GABA_A_ block (Bright and Smart, 2013). The mean values from the Gaussian fits revealed similar values to the mean current method that was thus used throughout the study.

### Drug applications

Drugs were applied to the slice perfusion for ≥5 min. Tetrodotoxin (TTX, 1 μM), NBQX (disodium salt, 10 μM), APV (50 μM), allopregnanolone (0.6 μM), and Bicuculline (25 μM) were from Hellobio (UK). Nipecotic acid (1 mM), picrotoxin (100 μM), THIP (1μM), CGP 52432 (5 μM), and kynurenic acid (2 mM) were from Abcam (UK).

### Immunofluorescence procedures

Mice were deeply anesthetized with isoflurane 5% and perfused transcardially with, in chronological order, phosphate saline buffer (PBS) in combination with heparin, PBS, and 4% PFA in 0.1 M PBS, pH 7.4. Brains were PFA fixed overnight, washed, and cut in 70 μm coronal or horizontal sections in PBS by vibratome (Leica Vibratome VT1000S), depending on the brain region of interest. First, floating sections were incubated for 1 h in the Blocking Serum (BS: 1% BSA, 2% goat serum, and 1% horse serum in PBS) and 0.1% TritonX-100. Then, primary antibodies were diluted in BS for anti δ and in BS 0.01% TritonX-100 for all the other antibodies (16 h at 4°C). Primary antibodies used were: anti-δ antibody (1:50 in rabbit, Sperk et al., 1997); anti-GFAP (RRID:AB_10013382, 1:300 in rabbit, Dako, Denmark, Z0334); anti- Iba1 (RRID:AB_10641962 1:1,000 in rabbit, Synaptic System); after washing with PBS, slices were incubated for 2 h at RT with specific secondary antibodies conjugated with Alexa Fluor-488, Alexa Fluor-546 1:500, Thermo Fisher Scientific Invitrogen). Nuclei were contra-stained by TopRo-3 (1:1,000, Invitrogen, Thermo Fisher Scientific). Floating sections were then washed and mounted on glass slides with an Elvanolmounting medium. Negative controls were performed in the absence of the primary antibodies. Immunofluorescence images were obtained with a Leica SP5 confocal microscope with a 20× objective. Notably, immunofluorescence experiments were always carried out on both control and Na_v_1.1^+^^/-^ brains of the same litter; images were acquired with the same parameters.

### Data analysis and statistics

Data analysis was performed with Clampfit 10, Origin 8.0 (Microcal Software), Microsoft Office. We used Student’s *t*-test or Mann Whitney for data that are or are not represented by a normal distribution (Shapiro-Wilk test), respectively. Results were considered statistically significant at *p* ≤ 0.05 (*p ≤ 0.05, **p ≤ 0.01, ***p ≤ 0.001). Immunofluorescence quantification: Confocal image z-stacks (8 μm, 1 μm step) with anti-δ antibodies were merged and used for 488 immunofluorescence quantification in ImageJ. In detail, field integrated density was corrected by background and related to the field area, for both DG and ECx of DS and control mice. For GFAP and Iba1 staining positive cell counting for field was evaluated.

## Results

**Figure 1:**
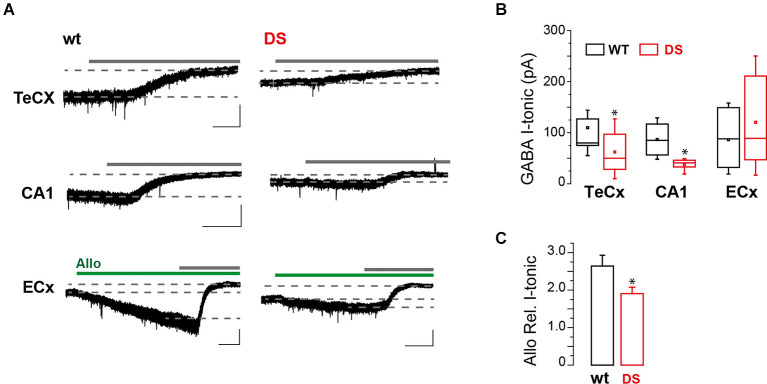
GABA tonic currents are altered in DS mice during epileptogenesis. **(A)** Whole-cell recordings showing representative GABA tonic currents (I-tonic) evoked by blocking all GATs with nipecotic acid in perfusion (not indicated) and unmasked with a GABA_A_ receptor antagonist (gray continuous lines; bicuculline or picrotoxin; see “Materials and methods” Section) in layer V pyramidal neuron of brain slices from 15 to 19 days old DS mice (Na_v_1.1^+^^/-^) and same age littermate wild type controls (wt). Dotted lines indicate the I-tonic before and after GABA_A_ receptor antagonist (gray bars; see “Materials and methods” Section). Green bars mark allopregnanolone (Allo) application. Scale bars, 1 min, 50 pA. **(B)** Box chart summarizing GABA I-tonic in wt and DS mice from TeCx (*n* = 9 cells from three mice for wt and 11 cells from five mice for DS mice, *p* = 0.042, two sample *t*-test), CA1 (*n* = 4 cells from four mice for wt and five cells from three mice for DS mice, *p* = 0.025, two sample *t*-test) and ECx (*n* = 7 cells from four mice for wt and nine cells from four mice for DS mice, *p* = 0.399, two sample *t*-test). **(C)** Histogram summarizing the relative GABA I-tonic potentiation in ECx (mean ± SEM; same cells as in **B**; *p* = 0.037, two sample *t*-test) induced by ALLO in wt and Na_v_1.1^+^^/-^ mice. *p ≤ 0.05, **p ≤ 0.01, ***p ≤ 0.001.

**Figure 2:**
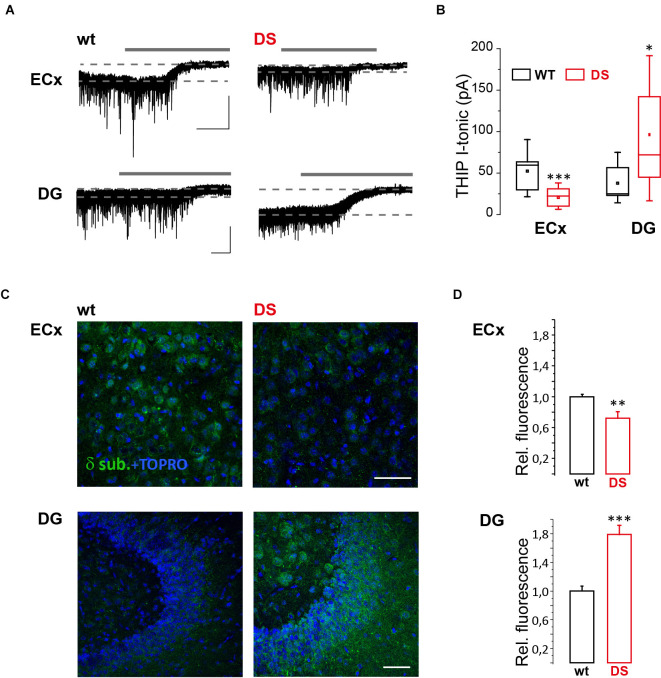
GABA_A_R δ subunit response and expression are altered in DS mice. ** (A)** Whole-cell recordings showing representative tonic currents (I-tonic) evoked by THIP (not indicated) and evaluated with GABA_A_ receptor antagonist bicuculline (gray continuous lines; see “Materials and methods” Section) in layer V pyramidal neuron of brain slices containing ECx and DG from 15 to 19 days old DS mice (Na_v_1.1^+^^/-^) and same age littermate wild type (wt) controls. Scale bars, 30 s, 50 pA. **(B)** Box chart summarizing I-tonic in DS and wt mice from ECx (*n* = 10 cells from four mice for wt and 10 cells from three mice for DS mice, *p* = 6.783 E^−4^, two sample *t*-test) and DG (*n* = 9 cells from five mice for wt and nine cells from five mice for DS mice, *p* = 0.017, two sample *t*-test). **(C)** Confocal fluorescence images of δ subunit expression in ECx and DG from 19 to 21 days old DS mice and same age littermate wt controls. **(D)** Histogram summarizing the relative fluorescence in DS compared to wt mice from ECx (26 and 29 fields from three wt and three DS mice, respectively, *p* = 0.003, two sample t test) and DG (27 and 38 fields from 4 wt and 4 DS mice, respectively, *p* = 0.00001, two sample *t*-test). *p ≤ 0.05, **p ≤ 0.01, ***p ≤ 0.001.

To study GABA tonic conductance in DS mice (Na_v_1.1^+^^/-^), we performed whole-cell patch-clamp recordings from principal neurons in brain slices obtained from PN15 to 19 animals, before the occurrence of overt spontaneous seizures, i.e., after P21 (Yu et al., 2006) or later than P22 (Oakley et al., 2009). We found that GABA tonic currents (see “Materials and methods” Section). are significantly reduced in the Temporal cortex (TeCx) and CA1 pyramidal neurons of DS mice compared to wild type controls (Figures 1A,B). Relative mean current densities in TeCx and CA1 region of DS mice were also significantly reduced to 0.62 ± 0.15 (*n* = 9 cells from three mice for wt and 11 cells from five mice for DS mice) and to 0.58 ± 0.14 (*n* = 4 cells from four mice for wt and five cells from three mice for DS mice) of controls (average of wild type for each litter; *p* = 0.035 for TeCx and *p* < 0.001 for CA1, one sample *t*-tests), respectively, confirming that in DS mice extrasynaptic GABA transmission is significantly reduced in these regions. In layer V neurons of the enthorinal cortex (ECx), a region strongly connected to the hippocampus, GABA tonic current was instead not significantly affected in DS mice compared to controls (Figures 1A,B; *n* = 7 cells from four mice for wt and nine cells from four mice for DS mice). Noteworthy in this region we observed that the effect of allopregnanolone (Allo), a potent endogenous neurosteroid with anticonvulsant effects (Biagini et al., 2010), was significantly smaller in DS mice compared to controls (Figures 1A,C). Since neurosteroids strongly potentiate extrasynaptic δ subunit-containing GABA_A_ receptors (Stell et al., 2003), we hypothesized a reduced function or expression of these receptors in DS mice. To directly evaluate this hypothesis, we evoked GABA tonic currents with 4,5,6, 7-tetrahydroisoxazolo (5,4-c) pyridin-3-ol (THIP; also known as gaboxadol), a selective agonist of δ subunit-containing GABA_A_ receptors. Whole-cell currents mediated by δ subunit activation were significantly reduced in ECx of DS mice compared to controls (Figures 2A,B; *n* = 10 cells from four mice for wt and 10 cells from three mice for DS mice) and the relative mean current density of DS mice was also reduced to 0.66 ± 0.14 of controls (*p* = 0.032 two samples *t*-test). We next tested THIP also in DG, a δ subunit-enriched region. Unexpectedly, in contrast to what we observed in ECx pyramidal neurons, we found that tonic currents were largely increased in DG granule cells of DS mice compared to controls (Figures 2A,B; *n* = 9 cells from five mice for wt and nine cells from five mice for DS mice). In immunofluorescence experiments, we then evaluated whether the altered THIP response that we observed is due to a change in subunit expression. Consistent with our electrophysiological data, δsubunit expression in DS mice was significantly reduced in ECx and increased in DG (Figures 2C,D) with respect to controls.

**Figure 3:**
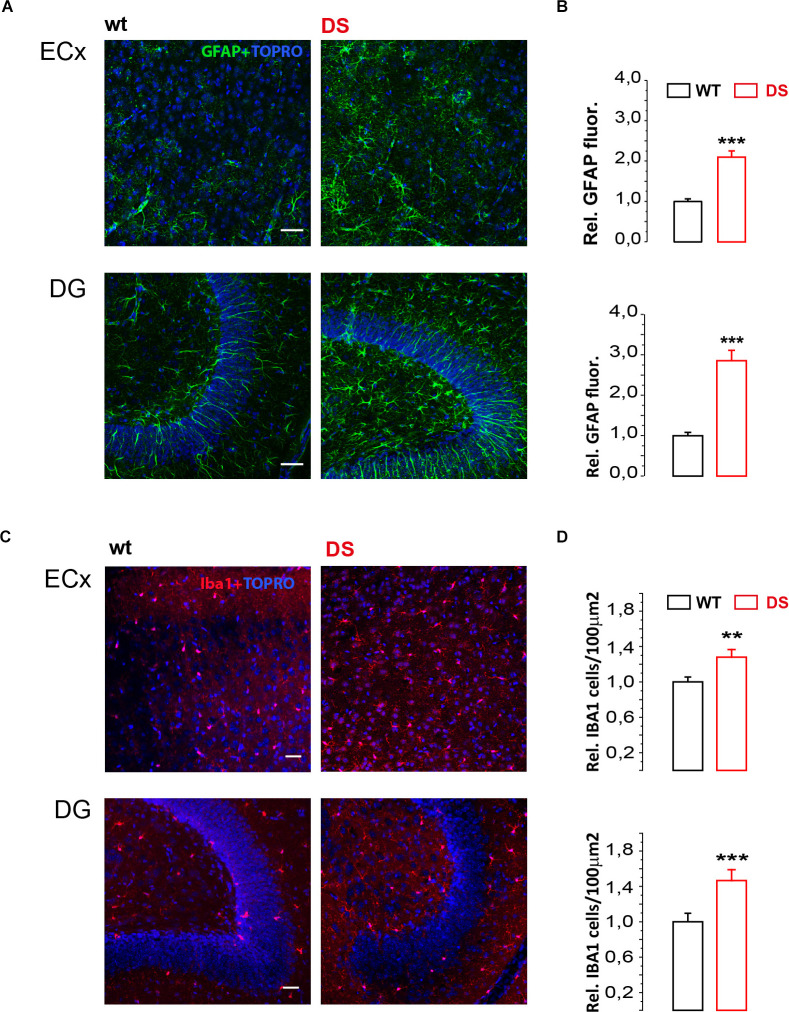
Glial cell markers GFAP and Iba1 are increased in DS mice. **(A,C)** Confocal fluorescence images of GFAP and Iba1 immunofluorescence in ECx and DG from 19 to 21 days old DS mice (Na_v_1.1^+^^/-^) and same age littermate wild type (wt) controls. **(B)** Histogram (mean ± SEM) summarizing the relative GFAP fluorescence in DS mice compared to wt mice from ECx (*n* = 3 mice PN 19–21, 6 ECx, 18 fields for WT and DS mice *p* < 0.001, one sample *t*-test) and DG (*n* = 3 mice PN 19–21, six DG, 18 fields for WT and DS mice *p* < 0.001, one sample *t*-test). **(D)** Histogram (mean ± SEM) summarizing number of Iba1 positive cells/μ^2^ in DS compared to WT mice from ECx (*n* = 4 mice PN 19–21, eight Ecx, 24 fields for WT and DS mice *p* = 0.00817, two sample *t*-test) and DG (*n* = 4 mice PN 19–21, eight DG, 24 fields for WT and DS mice *p* = 0.0038237, two sample *t*-test). **p ≤ 0.01, ***p ≤ 0.001.

Finally given that extrasynaptic GABA subunits are affected by several physiological and pathological events including inflammation (Roseti et al., 2015; Whissell et al., 2015; Crowley et al., 2016) and that reactive gliosis and neuroinflammation are frequently involved in childhood epilepsy (Koh, 2018), we evaluated microglia and astrocyte reactivity in DS mice before seizures onset. To this aim, we performed immunofluorescence experiments focusing on astrocytes and microglia reactivity in both ECx and DG. Our results reveal that GFAP expression is significantly increased in both DG and ECx of DS mice (*p* = 0.00001 for both; Figures 3A,B). In addition, Iba1 expression is also significantly increased in both DG (*p* = 0.005) and ECx (*P* = 0.008; Figures 3C,D). Therefore, during epileptogenesis DS mice show a significantly increased reactivity of glial cells.

## Discussion

In the present study, we focused on cellular and molecular changes during the epileptogenic period in a mouse model of DS, before overt spontaneous seizure appearance. Although DS is considered a multifocal epilepsy, studies reported a prominent role of the hippocampal region in this syndrome (Liautard et al., 2013; Stein et al., 2019; Mattis et al., 2021), with signs of hippocampal hyperexcitability being reported also before the epileptic period (Liautard et al., 2013). The reduced GABA tonic currents in TeCx and CA1 regions of DS mice that we here report may thus represent an important mechanism that leads to increased excitability in these brain regions of DS mice. In the ECx of DS mice, an area strongly connected to the hippocampus, we conversely found that the overall GABA tonic current is similar to controls but less responsive to allopregnanolone, an endogenous neurosteroid selective for δ subunits, suggesting altered GABA_A_ receptor subunit composition in this region. Indeed our electrophysiological data with a δ subunit selective agonist and quantitative immunofluorescence analysis revealed that δ subunit response and expression respectively are reduced in ECx of DS mice. Other GABA receptor subunits, such as α5, could compensate for this reduction, explaining the overall conserved GABA tonic response that we described, albeit with a different pharmacological profile. Surprisingly, in the DG, a region normally enriched in δ subunits and crucial for seizures in DS (Tran et al., 2020), response and expression of these subunits are strongly increased in DS mice compared to controls. These data reveal that during epileptogenesis of DS, δ subunit expression shows opposite alterations in two epileptogenic regions. Accordingly, the overall effect on brain excitability and seizure onset is not easily predictable. δ subunits are sensitive to different drugs/agents (anesthetics, barbiturates, ethanol) and to neurosteroids, a class of potent endogenous modulators of cellular and network excitability (Stell et al., 2003; Belelli et al., 2009). Neurosteroids, and neuroactive steroids in general, change in relation to physiological and pathological conditions, including catamenial epilepsy (Reddy and Rogawski, 2010) and other stress-related disorders such as anxiety, post-partum depression, and premenstrual syndrome (Whissell et al., 2015). Notably, stress alters neurosteroid levels and acute stress may induce seizures in DS patients (Verbeek et al., 2015), suggesting that hypothalamic–pituitary–adrenal axis and hormones levels are important for hyperexcitability and seizure onset in DS. Our data suggest that during epileptogenesis in DS a change in δ subunit-containing GABA receptors may affect the normal control of GABA tonic conductance and network excitability by endogenous neuroactive steroids. Future studies are needed to clarify whether the changes of δ subunit that we observed are protective, and their effect is insufficient or transient, or detrimental, and their effect may later induce seizures. Notably, the fact that GABAergic drugs used in DS to reduce seizures such as stiripentol, tiagabine, or SGE-516, either in therapy or in animal models (Grosenbaugh and Mott, 2013; Oakley et al., 2013; Hawkins et al., 2017), may increase GABA tonic conductance suggests that favoring this type of slow conductance is beneficial in DS. If so, the decreased overall GABA tonic currents in TeCx and CA1 and the reduction of δ subunit in ECx should favor tissue hyperexcitability and seizures, while increased δ subunits in DG could represent an insufficient protective mechanism.

The molecular mechanism that leads to altered extrasynaptic GABA_A_ receptor expression in DS also remains to be clarified. It is known that δ subunit expression is highly dynamic and may be altered in different physiological or pathological conditions including psychiatric disorders and epilepsy (Ferando and Mody, 2012; Whissell et al., 2015). One possibility is that that Na_v_1.1 haploinsufficiency and reduced excitability of GABAergic interneurons in developing DS mice may impair GABA release and extracellular concentration, affecting receptor expression by transcriptional or post translational receptor modulation (Jacob et al., 2008; Uusi-oukari and Korpi, 2010). The increased glial cell activation that we reveal in DS mice may also contribute to explaining the alteration of GABAergic receptors. Reactive astrocytes may, indeed, release GABA (Jo et al., 2014) and different neuroactive molecules including neurosteroids (Zwain and Yen, 1999) or cytokines, chemokines, and trophic factors (Verhoog et al., 2020; see also below) that could affect extrasynaptic GABA receptor composition. In addition, we may not rule out episodes of local network hyperactivity preceding overt spontaneous seizures that could locally affect both GABA subunits and glial cells locally. Astrocytes and microglia could also be affected directly by the impaired excitability of GABAergic cells that during development marks DS. Indeed GABA directly affects inflammatory pathways (Kuhn et al., 2004) and GABA agonists exert antiinflammatory properties by stimulating both GABA_A_ and GABA_B_ receptors on glial cells (Lee et al., 2011).

Finally, the opposite region-dependent changes of δ subunit expression that we found in ECx and DG may depend on local neuronal activity or glial heterogeneity that cannot be easily discriminated. Indeed astrogliosis comprises a spectrum of astrocytic changes affecting their transcriptome and secretome (Escartin et al., 2021) that, together with microglia, plays a crucial role in neuroinflammation. This latter is thought to take part in epileptogenesis through several, sometimes opposing, effects (Vezzani et al., 2019). Accordingly, our data suggest that altered glial cells may participate in DS development by acting on different possible signaling pathways or modulators. For instance, reactive astrocytes may have increased neurosteroidogenesis that could transiently protect from seizures during epileptogenesis, as revealed in another epilepsy model (Biagini et al., 2006). On the opposite, inflammation can reduce KCC2 transcription altering NKCC/KCC2 ratio, chloride homeostasis, and GABA control of membrane excitability (Pozzi et al., 2020) as previously described also in tissues from adult DS patients (Ruffolo et al., 2018). Finally activated glia may release different pro-inflammatory cytokines such as interleukin 1β (IL1-β) or tumor necrosis factor α (TNFα), danger signals like high mobility group box 1 (HMBG1), chemokines like CCL2–4, the transforming growth factor-β (TGFβ), the transcription factor nuclear factor erythroid 2-related factor 2 (NRF2), prostaglandins, reactive oxygen, or nitrogen species (ROS and RNS, respectively) and complement factors that, through several transcriptional and post-translational effects, ultimately induce alterations of the blood-brain barrier permeability, neuronal hyperexcitability and seizures (Rana and Musto, 2018; Vezzani et al., 2019; Verhoog et al., 2020; Granata et al., 2022). Whether and how these mechanisms take part in DS development needs further investigation, especially considering that febrile seizures, that characterize DS, involve many of the above-mentioned molecules and mechanisms.

In conclusion, our study reveals that during the development of DS, major changes take place in GABA_A_ extrasynaptic receptor composition and GABA tonic currents in parallel with an increase in microglia and astrocyte reactivity. The identification during DS development of the molecular mechanism on the basis of the functional interactions between activated glia and GABAergic system may reveal new targets for future interventions. Indeed growing evidence shows functional interactions between GABAergic signaling and inflammation (Crowley et al., 2016) as well as between glial cells and the maturation of the GABAergic system (Favuzzi et al., 2021). Overall, our data suggest that these functionally interconnected events, being altered, may represent a crucial step in DS development.

## Data Availability Statement

The raw data supporting the conclusions of this article will be made available by the authors, upon reasonable request.

## Ethics Statement

The animal study was reviewed and approved by Ministero della Salute and OPBA of Padua University.

## Author Contributions

RG performed patch-clamp recordings, immunofluorescence experiments, and data analysis. AC performed immunofluorescence experiments and data analysis. MG-G, IM, and LR performed patch-clamp recordings. PS provided delta subunit antibodies. GC wrote the manuscript with GL. GL performed patch-clamp recordings, conceived experiments, and wrote the manuscript with help from all authors. All authors contributed to the article and approved the submitted version.

## Conflict of Interest

The authors declare that the research was conducted in the absence of any commercial or financial relationships that could be construed as a potential conflict of interest.

## Publisher’s Note

All claims expressed in this article are solely those of the authors and do not necessarily represent those of their affiliated organizations, or those of the publisher, the editors and the reviewers. Any product that may be evaluated in this article, or claim that may be made by its manufacturer, is not guaranteed or endorsed by the publisher.
