# Corrigendum: GABA tonic currents and glial cells are altered during epileptogenesis in a mouse model of Dravet syndrome

**DOI:** 10.3389/fncel.2022.1066985

**Published:** 2022-10-20

**Authors:** Rosa Chiara Goisis, Angela Chiavegato, Marta Gomez-Gonzalo, Iacopo Marcon, Linda Maria Requie, Petra Scholze, Giorgio Carmignoto, Gabriele Losi

**Affiliations:** ^1^Department of Biomedical Science, University of Padua, Padua, Italy; ^2^Neuroscience Institute, National Research Council (IN-CNR), Padua, Italy; ^3^Department of Pathobiology of the Nervous System, Center for Brain Research, Medical University of Vienna, Vienna, Austria

**Keywords:** extrasynaptic GABA receptor A, delta subunits, GFAP - glial fibrillary acidic protein, Iba-1, SCN1A encephalopathy, Nav1.1 channel

In the published article, there was an error in the **Funding** statement. The “Euro Bioimaging Project Roadmap/ESFRI from European Commission” funding was accidentally omitted. The corrected **Funding** statement appears below.

## Funding

This study was funded by Gruppo Famiglie Dravet Onlus, Progetto di Ricerca 2017 to GL, European Union's Horizon 2020 research and innovation program EU-Glia PhD under the Marie Sklodowska-Curie grant agreement No. 722053 to GC, and Euro Bioimaging Project Roadmap/ESFRI from European Commission.

The authors apologize for this error and state that this does not change the scientific conclusions of the article in any way. The original article has been updated.

## Publisher's note

All claims expressed in this article are solely those of the authors and do not necessarily represent those of their affiliated organizations, or those of the publisher, the editors and the reviewers. Any product that may be evaluated in this article, or claim that may be made by its manufacturer, is not guaranteed or endorsed by the publisher.

